# The Nature and Symptoms of Exophthalmic Goitre

**Published:** 1894-03

**Authors:** A. Joffroy

**Affiliations:** Paris, France


ATLANTA

MEDICAL AND SURGICAL JOURNAL.

VOL. XI. MARCH, 1894. NO. 1.

EDITED BY

LUTHER B. GRANDY, M. D., AND MILLER B. HUTCHINS, M. D.,

WITH THE CO-OPERATION OF

H. V. M. MILLER, M. D., LL. D., VIRGIL O. HARDON, M. D.,

FLOYD W. McRAE, M. D., ARTHUR G. HOBBS, M. D.,

AND H. R. SLACK, PH. D.

### *ORIGINAL COMMUNICATIONS.*

### THE NATURE AND SYMPTOMS OF EXOPHTHALMIC GOITRE*.

BY M. LE DOCTEUR A. JOFFROY,
PARIS, FRANCE.

The greater number of doctors admit that exophthalmic goitre is a neurosis, but the arguments which lead them to this opinion appear to me either debatable or insufficient; for, in short, many facts which I have observed, and researches which I have undertaken, induce me think that Basedow's disease applies generally either to a functional vice, or to some alterations of the thyroid body more or less profound.

The pathology of exophthalmic goitre has been, until recently, entirely unknown, the description of the disease resolving itself into an association of a certain number of symptomatic pictures; symptoms observed on the part of the heart and blood vessels, the thyroid symptoms, the nervous symptoms, etc.

*A clinical lecture delivered at the *Hospice de la Salpetriere.* Translated from *Le Progrès Medical* by Dr. L. B. Grandy, Atlanta.

I will begin by a description of the goitre since I accord to the thyroid gland and its changes a preponderating rôle in the pathogenesis of Basedow's disease.

Here is a young woman, twenty-four years of age, who presents the greater part of the symptoms which one meets with in this affection. You will notice at once that she has a strange expression, not alone the prominent eyes, but she has something peculiar in her look which you will understand better in a moment, when we will study in detail the condition of her vision. A physician examining this patient would remark immediately the prominence of the eyes and would ask himself if he had not encountered an exophthalmic goitre. On inspecting at a glance the cervical region he would perceive a tumor occupying the sides and middle of the thyroid region.

With these two symptoms, prominent eyes and thyroid tumor, he would be able almost surely and without further examination to affirm the diagnosis of Basedow's disease.

Pursuing his investigations and counting the pulse, he would find about 120 or 130 beats per minute, and finally if he made the patient extend her hands he would observe a small vibratory tremor; in a word, he would notice at once the four principal signs which characterize the disease. But these are only a part of the symptoms of exophthalmic goitre, for this famous symptomatic trio is indeed increased, and there are, as you will see, other symptoms to study than the exophthalmia, the goitre and the tachycardia.

*Goitrous or hypertrophic changes of the thyroid body.*—With our patient we see that the tumor occupies the lateral and middle region. There are three lobes, and by palpation one appreciates an hypertrophy of the right and left lobes, and also the existence between these two lobes in the median line an additional tumor, large as a small orange, with an elastic resistance. You notice that the right lobe, conforming to an observation often made, is larger than the left.

On applying the entire hand to the tumor and exercising a certain compression one feels a peculiar sensation. The tumor is pulsatile, and is increased at each afflux of blood into the arteries, and at the same time under the ends of the fingers one feels a wellmarked thrill. The vascular element here plays an important rôle in the constitution of the tumor, but it is only one of the elements; the goitre entirely vascular exists only theoretically and is not demonstrated by necroscopic examinations. Very frequently one finds it well-marked in those cases where, following some great emotion, he sees after some days the neck to swell and to reach, perhaps, great proportions. It has been thought that this is only a distension of the thyroid body by an excessive afflux of blood, as if one had practiced a sort of injection into the organ under high pressure; in a word as if there was produced a veritable erection of the thyroid without lesion of the tissues.

It is not not necessary, I think, to make autopsies to show that this idea of a goitre entirely vascular is erroneous, and by deep palpation you will always detect some indurated nodules, or some portions of the organ harder than others which correspond either to some foci of inflammation or of colloidal infiltration.

Now, this alteration in the structure of the thyroid I have found in all goitres which I have examined. I have encountered some very vascular goitres; I have not found any without changes in structure, very easily appreciable by an attentive palpation of the tumor. I wish to emphasize the idea that every goitre which develops rapidly, even in a few hours, and in which the vascular element seems to be entirely and solely the cause, is a thyroid body previously altered in structure; the goitre purely vascular without other alteration of tissue does not exist.

The thyroid tumor, which is, when it exists, an important feature of Basedow's disease, may acquire, in certain cases, a considerable volume, even as large as the head of an infant, but generally it is much less developed. With the patient which I show you the tumor is of smaller dimensions; often the hypertrophy is still less, to the degree of being in reality no tumor at all, but only an increase in the volume of the thyroid. Sometimes, indeed, this hypertrophy of the organ is so little accentuated, so little apparent, that it escapes a simple inspection of the neck, and requires then to be searched for by a careful palpation. Thus the search for alterations of the thyroid body in this disease ought always to be very carefully made, not only because it is specially in women that exophthalmic goitre exists, and that these patients have a special talent for concealing all signs of the malformation, but also because in certain cases there is neither tumor nor, indeed, an hypertrophy visible to the eye, and a very precise palpation is necessary to detect the increase of volume. I will add, indeed, that the most careful examination may be insufficient and that it has happened to me to find at the autopsy a thyroid body which I had considered normal during life, and of which in reality the dimensions were more than double and the weight more than triple.

Here is, for example, a second patient who was placed in my service with a diagnosis of Basedow's disease without exophthalmia and without goitre. The diagnosis is entirely correct, the patient having neither exophthalmia nor goitre, but that does not say that the thyroid body is not altered. Indeed, you will observe by palpation that the left lobe is at least twice as large as the right, and the hypertrophy of this left lobe becomes clearly apparent when the patient, having slightly thrown her head back, makes a movement of deglutition.

To sum up, in Basedow's disease the goitre is perhaps considerable; more often of less dimensions; sometimes small; in some cases there is no tumor, there is only a certain degree of enlargement, but I repeat that palpation enables us to detect only considerable augmentations of volume. The point which I insist upon is that when indeed the thyroid body presents neither tumor nor apparent hypertrophy one cannot affirm that it is not altered.

During the course of this disease the size of the goitre may present very considerable fluctuations. Under the influence of emotion one sometimes sees it enlarge enormously; on the contrary, in other circumstances, with or without a remission of the other symptoms, the goitre may diminish or even disappear. At the time of the periods it is very frequent to see it increase in size. Finally some patients may be cured or very much improved in spite of a thyroid body very much enlarged.

### CARDIAC AND VASCULAR SYMPTOMS.

We now begin to study a group of symptoms which present themselves with remarkable constancy in Basedow's disease, those on the part of the heart and blood-vessels. The heart beats generally with energy, slowly and regularly. However, if the disease is of long standing and the patient cachectic, one will often observe some irregularity or even an intermission without there being any organic affection of the heart or its valves. But on this point patients differ. In some cases one sees after a long time some organic change in the heart and its valves added to the changes which had been purely functional. Finally, the heart disease and Basedow's disease may exist simultaneously as a mere coincidence.

### DILATATION OF THE ARTERIES.

Besides the cardiac symptoms which I have only indicated, we must note certain modifications of the peripheral circulation which are really very curious. The dilated carotids beat violently, and the same phenomenon may be noticed in the head. One may see it also in the upper part of the aorta, behind the sternum; sometimes it is observed in the fundus of the eye by the ophthalmoscope, but ordinarily it is found only in the cervical and cephalic regions, and not in the rest of the body. Thus, after feeling the carotid pulse one finds an artery large and dilating itself at the moment of systole, while the radial pulse is small, perhaps scarcely appreciable.

### OCULAR SYMPTOMS.

*Exophthalmia.*—The exophthalmia, which is the most important ocular symptom, is frequent, but far from being constant. The second patient which I showed you does not have it. When this sign exists it is symmetrical; almost always it is more pronounced on one side. Thus with our first patient the left eye is more prominent than the right. There is no established relation between the degree of the exophthalmia and that of hypertrophy of the thyroid, nor between the side of the exophthalmia and that of the goitre. Thus you may observe a tumor of the thyroid absolutely unilateral with a double exophthalmia, or a unilateral exophthalmia with a unilateral goitre on the other side.

In many cases there is no disturbance of vision in spite of considerable prominence of the eyeball. With our first patient there was no such disturbance. In other cases there is visual trouble. Our second patient is very myopic, but she has no exophthalmia, and her myopia existed before her Basedow's disease. Sometimes there will be a photophobia, requiring the patient to dwell in a dark chamber. Other patients may have diplopia.

We do not see any pathological modification in the internal musculature of the eye; the muscles of the iris and of accommodation are intact or are involved only accidentally. But it is not so with the motor muscles of the globe, or eyelid, or eyebrow, nor with those of the forehead and face. Here we observe some very curious symptoms which are of recent knowledge.

*Sign of de Graefe.*—I believe that it is to these motor troubles to which I have alluded that is due the symptom of de Graefe which has never been well understood. [This symptom is] that the upper eyelid, which in a normal state ought to follow and move with the ball, as if the lid and ball were more or less united, ceases to have this parallel motion with the globe, and the one remains behind the other. I think that this sign is due either to some diminution of mobility of the ball by which it is not able to follow the sound lid, or to a certain degree of paralysis or localized contracture of the upper lid. Thus, with our patient, you can easily see that it is the movement of the globe which is interfered with; and if we ask her to look up, the upper lid disappears under the brow while the ball executes a slow and limited movement upward.

*Sign of Stellwag.*—Besides this sign, there are others which show that the muscles in the neighborhood of the eye may also be affected. First, I will speak of the sign of Stellwag, characterized by an enlargement of the palpebral fissure when the eye is open, and its incomplete occlusion when the subject believes that the eyes are tightly closed. We need not believe that this is a consequence of the exophthalmia, and that this phenomenon is due to any insufficiency of the lid which renders it unable to cover the enlarged ball; because the sign of Stellwag may be absent with an exaggerated exophthalmia, and, on the other hand, we meet with it frequently when the exophthalmia is very much reduced, or even absent. I have now under my care a young girl who has no exophthalmia, and in whom the sign of Stellwag exists very plainly on the right side. The palpebral fissure is habitually greater on that side, and when she is asked to close her eyes the borders of the lids approach to the point of contact on the left side, while on the right they remain separated at least two millimeters, and, consequently, do not entirely cover the ball. There is on that side a paralysis of the muscles of occlusion.

[M. Joffroy described further a "*batting*" *of the lids*, a vibratory motion analogous to that sometimes observed in hysteria. Also he had seen in a number of cases *paralysis of the muscles of the forehead* and *facial paralysis*. These paralytic phenomena, he says, are almost always double, but they may be unilateral, or only slightly noticeable on one side.—Tr.]

The above paralyses play a very important rôle in Basedow's disease, and yet they have been very little studied up to the present time.

